# Clinical Predictive Factors of Lower Extremity Deep Vein Thrombosis in Relative High-Risk Patients after Neurosurgery: A Retrospective Study

**DOI:** 10.1155/2020/5820749

**Published:** 2020-06-04

**Authors:** Juhua Li, XinZhen Ren, Xiaole Zhu, Huayu Chen, Zhen Lin, Mei Huang, Zejuan Gu

**Affiliations:** ^1^Department of Neurosurgery, The First Affiliated Hospital of Nanjing Medical University, Nanjing, China; ^2^Department of Nursing, The First Affiliated Hospital of Nanjing Medical University, Nanjing, China; ^3^Pancreatic Center & Department of General Surgery, The First Affiliated Hospital of Nanjing Medical University, Nanjing, Jiangsu, China

## Abstract

**Introduction:**

It is acknowledged that patients undergoing neurosurgery with neurological illness are at higher risk of lower extremity deep vein thrombosis (DVT). As an underlying life-threatening complication, the incidence and risk factors for high-risk patients with lower extremity deep vein thrombosis are still controversial in relative high-risk patients after neurosurgery.

**Materials and Methods:**

A total of 204 patients who underwent neurosurgery and were considered as a high-risk group of DVT according to times of stay in bed more than 3 days were enrolled in this study. We evaluated the lower extremity DVT by using Color Doppler Ultrasound System (CDUS). Clinical parameters of patients at the time of admission and postoperation were recorded and prepared for further analysis. Early predictive factors for postoperative lower extremity DVT were established. Diagnostic performance of predictive factors was evaluated by using receiver operating characteristic (ROC) curve analysis.

**Results:**

The overall incidence rate of DVT in 204 enrolled patients was 30.9%. Multivariate logistic regression indicated that hypertension (OR 3.159, 95% CI 1.465-6.816; *P* = 0.003), higher postoperative D-dimer (OR 1.225, 95% CI 1.016-1.477; *P* = 0.034), female (OR 0.174, 95% CI 0.054-0.568; *P* = 0.004), and lower GCS score (OR 0.809, 95% CI 0.679-0.965; *P* = 0.013) were independently associated with incidence of DVT in patients after neurosurgery. The logistic regression function (LR model) of these four independent risk factors had a better performance on diagnostic value of DVT in patients after neurosurgery.

**Conclusion:**

The combined factor was constructed by hypertension, postoperative D-dimer, gender, and GCS score, and it might be a more handy and reliable marker to stratify patients at risk of DVT after neurosurgery.

## 1. Introduction

Deep vein thrombosis (DVT) is defined as formation of a thrombus within a deep vein and often caused by hypercoagulation and vascular endothelial injury [1]. Each year, over 550,000 hospitalized patients were affected by DVT in the United States, and DVT might cause a serious financial burden on patients [2, 3]. DVT is considered a common vascular disease in patients and accounts for 30% cases of pulmonary embolism (PE) [4]. As a life-threatening complication, mortality rate in patients with pulmonary thrombosis is about 14% [5]. It is important to recognize patients at risk for DVT early as they may require antithrombotic and thrombolytic therapy.

For surgical patients, those who undergo neurosurgery are considered to be much more susceptible to DVT [6]. Statistics suggest that incidence of venous thrombosis complications in neurosurgery patients varies from 1.7 to 34%; researches further indicated that brain malignant tumor and limb plegia of prolonged duration could increase the risk of venous thrombosis [7–9]. Patients with DVT usually present with a mild form. However, a higher overall mortality in about 6% of patients with DVT was recorded. While about 30% of patients with DVT develop into pulmonary embolism, overall mortality even exceeds 10% [4, 10]. As an effective measure, early initiation of heparin treatment is recommended for prevention of DVT [11]; however, it is still controversial, especially in patients with a higher risk of bleeding. Although several previous studies have identified that old age, restricted movement, overweight, cancer, pregnancy, and surgery are risk factors for DVT, the simple and accurate biomarkers for DVT in patients are still needed. Early screening of the DVT in neurosurgery patients is very necessary for lower morbidity and mortality. Evaluation of risk factors of DVT for neurosurgery patients is necessary for postoperative more aggressive mechanical and chemical prophylaxis.

## 2. Materials and Methods

### 2.1. Patients and Study Design

**Figure 1:**
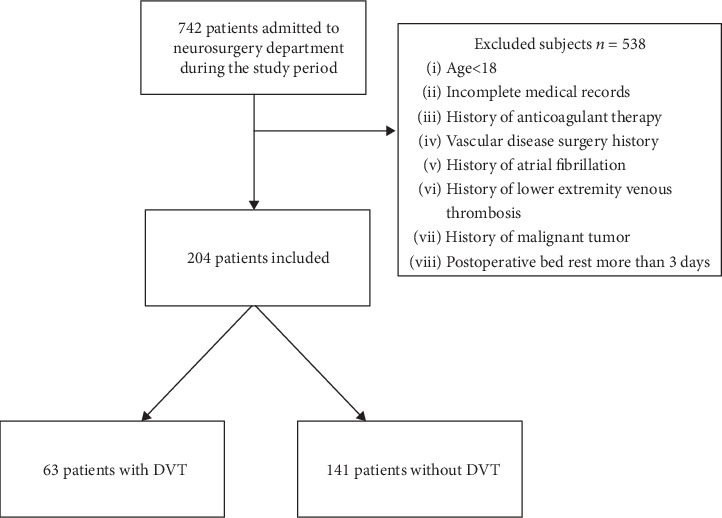
Study flow diagram.

This is a retrospective observational study and approved by the Institutional Review Board of the First Affiliated Hospital of Nanjing Medical University; patients who underwent neurosurgery admitted to the First Affiliated Hospital of Nanjing Medical University (Nanjing City, Jiangsu Province, China) from January 1 to December 31, 2018, were included in the study cohort. Informed consent from individual participants was obtained. As shown in Figure 1, of 742 patients, 538 patients were excluded from the study cohort for one or more of the following reasons: (1) age < 18 years old; (2) incomplete medical records; (3) history of anticoagulant therapy; (4) vascular disease surgery history; (5) history of atrial fibrillation; (6) history of lower extremity venous thrombosis; (7) history of malignant tumor; and (8) postoperative bed rest more than 3 days. Utilization of mechanical and physical therapy was applied to avoid DVT except for anticoagulant therapy in patients before or after surgery. Relative high-risk patients were defined as people who had bed rest more than 3 days after neurosurgery.

All enrolled patients were grouped into a DVT group or non-DVT group according to whether there is a DVT by duplex ultrasonography examination. All clinical characteristics of enrolled patients such as gender, age, BMI (body mass index), hypertension, pathological type, bedridden time, past medical history, and preoperative and postoperative D-dimer serum levels were retrieved from medical records. An electronic health medical record system and paper-based medical record library which allows access to more detailed information were noted in this study.

### 2.2. Diagnosis of DVT

Autar DVT risk assessment scale was an accurate and reliable indicator for DVT，and it was applied for the risk assessment of thrombosis in both groups on the day before surgery. Both lower extremities were confirmed with DUS (Vinno, Suzhou, China) by two different experienced ultrasound doctors preoperation and on postoperative days 1, 3, 6, 9, and 14.

### 2.3. Statistical Analysis

All data were performed in SPSS17.0 software (SPSS Inc., IBM Corporation, USA) and Stata/SE version 10.0 for Windows. Results were presented as mean ± standard deviation for continuous variables and frequencies (percentages) for categorical variables. The comparison of continuous variables was performed by Student's *t*-test between the two groups, and chi-squared test or Fisher's exact test was used to compare categorical variables. Univariate logistic regression models were used to examine the association between potential risk factors and DVT, and the multivariate logistic regression analysis was performed to determine the independent risk factors for DVT in neurosurgery patients. A two-tailed *P* value less than 0.05 was used to indicate statistical significance. Receiver operating characteristic (ROC) curve analysis was performed to examine the predictive role of factors for the diagnosis of DVT and to determine the cutoff values.

## 3. Results

### 3.1. Demographic and Clinicopathologic Characteristics

In the study period, a consecutive series of 742 patients suffering from neurological illness attended to the Department of Neurosurgery, the First Affiliated Hospital of Nanjing Medical University, from January 1 to December 31, 2018. According to inclusion criteria (Figure 1), as the baseline characteristics of these 204 patients were summarized in Table 1, 204 patients with an average age of 57.8 ± 11.99 years were included for analysis, of which 118 (57.8%) were male, 38 (18.6%) patients had a history of smoke, 33 (16.2%) patients had a history of drink, and 81 (39.7%) patients had hypertension. There were 105 (51.5%) patients who had surgery because of malignant tumor. Overall length of hospital stay was 19.76 ± 7.60 days. Paresis accounted for 103 (50.5%) of the total cases. Among the enrolled 204 patients who underwent neurosurgery, a total of 63 patients were diagnosed with DVT after neurosurgery in our hospital. Among the two groups (DVT and non-DVT group), the DVT group had an older age (60.4 ± 11.68 vs. 56.7 ± 11.99, *P* = 0.039) and longer length of hospital stay (21.5 ± 9.17 vs. 19.0 ± 6.68, *P* = 0.031) compared with the non-DVT group. Female patients (36/63, *P* = 0.006) seem to be more prone to DVT after neurosurgery. Additionally, patients in the DVT group had a higher proportion of paresis (40/63, *P* = 0.015), hypertension (36/63, *P* = 0.001), and malignancy (40/63, *P* = 0.032). Higher preoperative (3.7 ± 5.19 vs. 1.1 ± 1.83, *P* < 0.001) and postoperative D-dimer (5.4 ± 6.65 vs. 1.3 ± 2.23, *P* < 0.001) levels were found in the DVT group than in the non-DVT group.

### 3.2. Univariate Logistic Regressions

To assess the independent predictors of DVT in patients after neurosurgery, univariate logistic regression analysis was performed and the results (Table 2) revealed that the risk of DVT significantly increased in patients with older age (OR 1.028, 95% CI 1.001-1.055; *P* = 0.041). Meanwhile, female patients seemed to be one of the risk factors for DVT in neurosurgery patients (OR 0.412, 95% CI 0.225-0.756; *P* = 0.004). Significant predictive factors for DVT after neurosurgery patients also included lower Glasgow Coma Scale score (GCS score) (OR 0.412, 95% CI 0.225-0.756; *P* = 0.004), hypertension (OR 2.844, 95% CI 1.542-5.245; *P* = 0.001), lower height (OR 0.949, 95% CI 0.912-0.987; *P* = 0.009), higher preoperative (OR 1.300, 95% CI 1.146-1.476; *P* < 0.001) and postoperative D-dimer (OR 1.354, 95% CI 1.202-1.524; *P* < 0.001), longer length of hospital stay (OR 1.043, 95% CI 1.003-1.084; *P* = 0.037), and higher Autar score (OR 3.365, 95% CI 2.371-4.774; *P* < 0.001).

### 3.3. Multivariate Logistic Regression

To ascertain the independent risk factors of DVT in patients after neurosurgery, potential predictors with*P* < 0.1in univariate logistic regression analysis were included in a multivariate logistic regression (Table 3). Autar score was ruled out in the multivariate analysis due to the fact that it consists of age, BMI, major surgery, medical disease, etc.; it may have an impact on our exploration of new predictors. By multivariable analysis, results show that female (OR 0.174, 95% CI 0.054-0.568; *P* = 0.004) and hypertension (OR 3.159, 95% CI 1.465-6.816; *P* = 0.003) were positively and independently associated with DVT in patients after neurosurgery. Moreover, GCS score was negatively related to DVT (OR 0.809, 95% CI 0.679-0.965; *P* = 0.013), and higher postoperative D-dimer was associated with an increased rate of DVT in patients after neurosurgery (OR 1.225, 95% CI 1.016-1.477; *P* = 0.034).

### 3.4. Diagnostic Value of Independent Risk Factors

**Figure 2:**
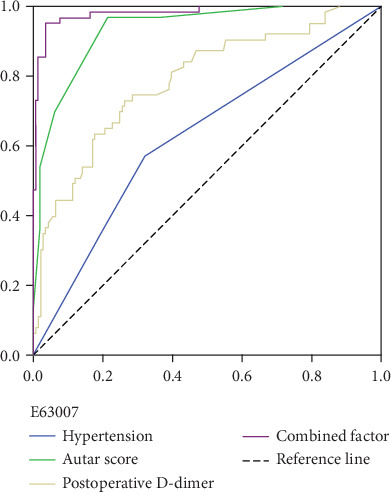
Comparisons of the AUCs between individual independent risk factors and combined factors in prediction of DVT.

**Figure 3:**
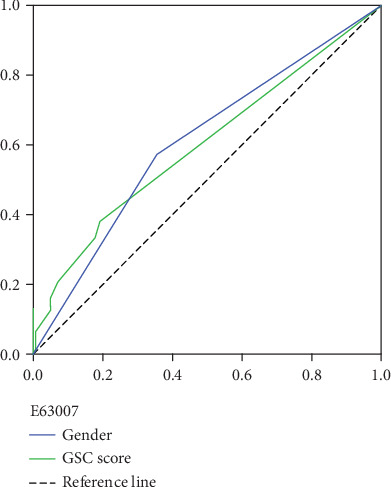
Receiver operating characteristic (ROC) curve for gender and GCS score in prediction of DVT.

An ROC curve analysis was conducted to evaluate the DVT diagnostic value of independent risk factors in patients after neurosurgery. The area under the ROC curves (AUC) and optimal cutoff values were calculated and shown in Tables 4 and 5 and Figures 2-3. The ability of hypertension (AUC 0.63, 95% CI 0.553-0.699; *P* < 0.001), gender (AUC 0.61, 95% CI 0.535-0.682; *P* = 0.004), GCS score (AUC 0.60, 95% CI 0.528-0.672; *P* = 0.006), and postoperative D-dimer (AUC 0.79, 95% CI 0.718-0.857; *P* < 0.001) to predict DVT in patients after neurosurgery is good. However, when compared with Autar score (AUC 0.93, 95% CI 0.899-0.966; *P* < 0.001), their diagnostic value is not significant. To build a simple and efficient diagnostic factor for DVT in patients after neurosurgery, these four independent risk factors were selected for combination (hypertension+postoperative D-dimer+gender+GCS) by regression coefficients. The new combined factor (AUC 0.98, 95% CI 0.963-0.999; *P* < 0.001) had a higher AUC, sensitivity (95%), and specificity (97%) for DVT than the diagnostic value of Autar score, and it was a reliable predictive index for DVT in patients after neurosurgery.

## 4. Discussion

Currently, it has been reported that postoperative patients are at significantly increased risk of DVT especially in neurosurgical patients. Prior studies had suggested that incidence of DVT in neurosurgical patients varies from 6% to 43% [12]. As a result of lacking specific symptoms, DVT is often ignored and misdiagnosed in the clinical work. DVT is an important cause of prolonged inpatient stays and unexpected death in hospitalized patients; however it is considered a preventable complication. DVT of the lower extremity in neurosurgical patients can be caused by various factors both in genetic and in acquired factors [13, 14]. Our study was designed to investigate the incidence of DVT and identify potential predictors for DVT in patients after neurosurgery.

In neurosurgical patients, there were still no general guidelines and uniform standards for preventing DVT, though nursing mechanical intervention and pharmacologic anticoagulant treatment have been confirmed to effectively reduce DVT. However, pharmacologic prophylaxis such as heparin is not widely accepted in neurosurgical operation because of possible intracranial hemorrhage. How to select early anticoagulant treatment and nursing mechanical intervention on relative high-risk patients after neurosurgery is still unclear. According to the recent American College of Chest Physicians (ACCP) 2012 guidelines, mechanical prophylaxis is preferred over pharmacological prophylaxis in routine craniotomy patients. The most common mechanical methods for DVT prophylaxis are compression stockings (CS) and intermittent pneumatic compression (IPC) of the lower extremities [15]. This study was conducted to screen strategy for the detection of DVT in neurosurgical patients, and a more targeted preventive measure should be taken for relative high-risk groups.

The findings of this study showed that the incidence of DVT was 30.9% in patients who kept in beds more than 3 days after neurosurgery during hospitalization. Univariate and multivariate analysis results indicated that female correlated with DVT risk. Previous studies revealed that men have a slightly higher overall incidence rate than women, whereas during the reproductive years, women have a slightly higher rate [16]. An appearance of a hypofibrinolytic state was observed in pregnancy period. At that time, coagulation factors were increased including fibrinogen, factor VIII, and factor von Willebrand and inhibitors such as protein S were decreased, while taking hormonal contraceptives and hormone replacement therapy can also increase the risk of DVT in women after neurosurgery. Further information of enrolled women should be gathered for further analysis.

Despite the use of serum D-dimer, it seemed to be a reliable marker for the detection of DVT. But a variety of conditions might increase the D-dimer level including hemorrhage, infection, and trauma; what is more, there is no set value of D-dimer that implies the occurrence of DVT. In our study, despite the fact that both preoperative D-dimer and postoperative D-dimer were statistically higher in DVT-positive patients, only postoperative D-dimer was an independent risk factor for DVT in patients after neurosurgery. Our results indicated that a high value of preoperative D-dimer does not indicate the existence of DVT. Detection value of postoperative D-dimer is important.

The results presented here also found that a GCS score less than 5 was an independent risk factor for DVT. GCS includes eye opening, verbal, and motor responses, which was used ubiquitously in acute care databases and serves as an assessment tool for acute neurologic injury [17–19]. The present study suggested that a lower GCS score should be paid more attention in patients after neurosurgery. Multivariable models suggested that hypertension was also an independent risk factor of DVT in neurosurgical patients. Hypertension has been verified as a risk factor of DVT previously [20]. Several prospective studies have suggested that hypertension patients have a 2-fold increased likelihood of developing DVT [21]. We came to the same conclusion in neurosurgery patients.

Autar score was widely used as a clinical scoring system for DVT, However, this scoring system was not only for neurosurgery patients, and a large number of parameters were required for calculation, which makes it clinically cumbersome. We kept the four variables (hypertension, postoperative D-dimer, gender, and GCS) in a new model for prediction of DVT in neurosurgery patients. The final parameter calculation was based on regression coefficients. Based on the ROC curve analysis, the combined factors achieved higher AUC, compared with Autar score performed. We considered that it was easy to use for prediction of DVT in neurosurgery patients.

There were also some potential limitations that should be acknowledged in this study, such as relatively small sample size, single-center study, and retrospective nature. These limitations might lead to several biases and false conclusions. In addition, a well-designed, prospective study with more data will be needed to validate the results of this study.

## 5. Conclusion

In conclusion, we found that hypertension, postoperative D-dimer, gender, and GCS score were independently associated with incidence of DVT in patients after neurosurgery. Compared to Autar score, the combination of these four independent risk factors had a better performance on the diagnostic value of DVT in patients after neurosurgery, and it could be a more handy and reliable marker to improve clinical care and management strategies in patients after neurosurgery. Meanwhile, additional studies and statistics are required to further validate our results.

## Figures and Tables

**Table 1:** Comparison of demographic data.

Patients	Total (*n* = 204)	DVT(+)*n* = 63	DVT(-)*n* = 141	*P* value
Gender				
Male	118 (57.8%)	27	91	0.006
Female	86 (42.2%)	36	50	
Hypertension				
Yes	81 (39.7%)	36	45	0.001
No	123 (60.3%)	27	96	
Pathology				
Malignant	105 (51.5%)	40	65	0.032
Benign	99 (48.5%)	23	76	
History of drink				
Yes	33 (16.2%)	12	21	0.537
No	171 (83.3%)	51	120	
History of smoke				
Yes	38 (18.6%)	10	28	0.564
No	166 (81.4%)	53	113	
Paresis				
Yes	103 (50.5%)	40	63	0.015
No	101 (49.5%)	23	78	
Bedridden more than 7 days				
Yes	87 (42.6%)	31	56	0.223
No	117 (57.4%)	32	85	
BMI	23.7 ± 2.92	24.5 ± 3.39	23.4 ± 2.62	0.020
Age	57.8 ± 11.99	60.4 ± 11.68	56.7 ± 11.99	0.039
D-dimer (ng/ml)				
Preoperative	1.9 ± 3.46	3.7 ± 5.19	1.1 ± 1.83	<0.001
Postoperative	2.6 ± 4.55	5.4 ± 6.65	1.3 ± 2.23	<0.001
Length of hospital stay	19.76 ± 7.60	21.5 ± 9.17	19.0 ± 6.68	0.031
Autar score				<0.001
≤10	113	2	111	
10-14	75	46	29	
≥15	16	1	1	

**Table 2:** Univariate logistic regression analysis.

	N	Odds ratio	Std. Err.	z	*P* value	LOR	UOR
Gender	204	0.4120879	0.1275502	-2.86	0.004	0.2246593	0.7558845
Age	204	1.027768	0.0137985	2.04	0.041	1.001076	1.055171
Paresis	204	1.167675	0.1838017	0.98	0.325	0.8577009	1.589674
GCS score	204	0.8298877	0.0525759	-2.94	0.003	0.7329818	0.9396054
Bedridden	204	1.470424	0.4487322	1.26	0.206	0.8084996	2.674271
History of smoke	204	0.7614555	0.3078313	-0.67	0.5	0.3447719	1.681734
History of drink	204	1.344538	0.5359523	0.74	0.458	0.6155619	2.9368
Hypertension	204	2.844444	0.8879665	3.35	0.001	1.54267	5.244715
Height	204	0.9488808	0.0191477	-2.6	0.009	0.9120845	0.9871616
Weight	204	1.008087	0.0169047	0.48	0.631	0.9754933	1.041771
BMI	204	1.149204	0.062978	2.54	0.011	1.032168	1.279512
Autar score	204	3.364549	0.6006181	6.8	<0.001	2.371241	4.773952
Length of hospital stay	204	1.042662	0.0208805	2.09	0.037	1.00253	1.084401
Preoperative D-dimer	204	1.300772	0.0836553	4.09	<0.001	1.146723	1.475515
Postoperative D-dimer	204	1.353909	0.0818205	5.01	<0.001	1.202677	1.524158

**Table 3:** Multivariate logistic regression analysis.

	N	Odds ratio	Std. Err.	z	*P* value	LOR	UOR
Postoperative D-dimer	204	1.224775	0.1169895	2.12	0.034	1.015664	1.476938
Preoperative D-dimer	204	1.165525	0.1312309	1.36	0.174	0.9347201	1.453321
Gender	204	0.174473	0.105112	-2.9	0.004	0.0535696	0.5682483
GCS score	204	0.8094814	0.0724066	-2.36	0.018	0.6793107	0.9645957
Hypertension	204	3.159971	1.239301	2.93	0.003	1.46505	6.815752
Height	204	1.0078	0.037122	0.21	0.833	0.937607	1.083249
BMI	204	1.10445	0.0763342	1.44	0.151	0.9645283	1.264669
Length of hospital stay	204	1.033412	0.0300887	1.13	0.259	0.9760901	1.0941
Age	204	1.036752	0.0192954	1.94	0.052	0.999615	1.075268

**Table 4:** Discriminatory ability of independent risk factors for DVT in patients after neurosurgery.

Marker	AUC	SE.AUC	Lower limit	Upper limit	z	*P* value	Cutoff
Hypertension	0.62614	0.03709	0.55345	0.69883	3.4011	0.00067	1
Autar score	0.93291	0.01709	0.8994	0.96641	25.32387	<0.001	11
Postoperative D-dimer	0.78723	0.03544	0.71777	0.8567	8.10402	<0.001	1.23
Combined factor	0.98165	0.00913	0.96375	0.99955	52.72708	<0.001	0.43
Gender	0.60841	0.03737	0.53517	0.68164	2.90133	0.00372	0
GSC score	0.59974	0.03662	0.52798	0.67151	2.72404	0.00645	5

**Table 5:** Corresponding sensitivity and specificity.

	Sensitivity	Specificity	Positive predictive value	Negative predictive value	Positive likelihood ratio	Negative likelihood ratio
Autar score	0.97 (0.89, 0.99)	0.79 (0.71, 0.85)	0.67 (0.57, 0.95)	0.98 (0.94, 0.99)	4.55 (3.30, 6.27)	0.04 (0.01, 0.16)
Combined factor	0.95 (0.87, 0.99)	0.97 (0.92, 0.99)	0.92 (0.83, 0.98)	0.98 (0.94, 0.99)	26.8 (11.3, 63.6)	0.05 (0.02, 0.15)
GSC score	0.38 (0.26, 0.51)	0.81 (0.73, 0.87)	0.47 (0.37, 0.60)	0.75 (0.63, 0.82)	1.99 (1.25, 3.16)	0.77 (0.62, 0.94)
Gender	0.57 (0.44, 0.69)	0.65 (0.56, 0.72)	0.42 (0.34, 0.55)	0.77 (0.67, 0.83)	1.61 (1.18, 2.19)	0.66 (0.49, 0.91)
Hypertension	0.57 (0.44, 0.69)	0.68 (0.60, 0.76)	0.44 (0.36, 0.58)	0.78 (0.68, 0.84)	1.79 (1.29, 2.47)	0.63 (0.46, 0.86)
Postoperative D-dimer	0.73 (0.60, 0.83)	0.74 (0.66, 0.8)	0.55 (0.46, 0.69)	0.86 (0.78, 0.90)	2.78 (2.03, 3.81)	0.37 (0.24, 0.56)

## Data Availability

All data in our study are available from the corresponding authors upon reasonable request.
